# 3,4-Methylenedioxymethamphetamine (MDMA)-Assisted Therapy in Hawaii: A Brief Review

**DOI:** 10.7759/cureus.26402

**Published:** 2022-06-28

**Authors:** Ann Inouye, Aaron Wolfgang

**Affiliations:** 1 Psychology, Hawaii School of Professional Psychology, Honolulu, USA; 2 Psychiatry, Brooke Army Medical Center, San Antonio, USA

**Keywords:** psychoactive drug, hawaii, psychedelics, trauma, neuropsychology, neurobiology, mdma-assisted psychotherapy, mdma-at

## Abstract

The Food and Drug Administration (FDA) granted breakthrough therapy status to 3,4-methyl​enedioxy​methamphetamine-assisted therapy (MDMA-AT) in 2017 due to preliminary evidence supporting its efficacy and safety in treating post-traumatic stress disorder (PTSD). A series of six phase-II clinical trials studying MDMA-AT for treatment-resistant PTSD found that 54% of MDMA-AT full-dose participants no longer met the diagnosis of PTSD after two MDMA sessions, compared to 23% in the control group. In the first phase-III clinical trial, 67% no longer met the criteria for PTSD after three sessions. The effects are durable, with 67% no longer diagnosable after one year and 74% at nearly four years. The MDMA-AT is being fast-tracked for potential FDA approval by 2023. In 2021, Hawaii’s Senate Bill 738 unsuccessfully proposed that psilocybin be removed from the Schedule I controlled substances list due to its clinical efficacy for major depressive disorder. Methyl​enedioxy​methamphetamine is also a Schedule I controlled substance and has proven to be a treatment option that could potentially benefit the people of Hawaii.

## Introduction and background

Of Hawaii's residents, around 8.8% and 8% report a lifetime prevalence of depression or anxiety, respectively [1]. A Hawaii personality and health survey showed that 83.1% of participants reported at least one traumatic event occurring in their lifetime [2], higher than the national average of 70.4% [3]. Yet around 20% of Hawaii's residents, exceeding the national average of 8.3%, were not able to receive care due to access to care barriers [1].

On April 4, 2019, United States Senator Brian Schatz (D-HI) wrote a letter to the Food and Drug Administration (FDA) and the National Institute of Health (NIH) inquiring about the government’s plans to investigate the medicinal value of psychedelics [4]. On June 13, 2019, the FDA and NIH responded in a co-authored message acknowledging the therapeutic potential of psychedelics from the results of recent clinical trials and the need for more research [5]. On June 28, 2021, the Multidisciplinary Association for Psychedelic Studies (MAPS) responded to the FDA and NIH with clarification regarding clinical trials evidence supporting the safety and efficacy of psychedelics in the treatment of mental health disorders [5].

On January 22, 2021, Hawaii Senator Stanley Chang (District 9) introduced Senate Bill No. 738, which will potentially remove psychedelic mushrooms (psilocybin) from the list of Schedule I controlled substances and establish psychedelic treatment centers as well [6]. Promising clinical trials of psychedelics used as medications to treat mental health disorders, such as major depressive disorder, are driving the State of Hawaii to consider the potential medical benefits of psychedelics for Hawaii's residents [4].

Ketamine, a substance producing psychedelic experiences and hallucinations, has been used to treat depression for many years. In 2019, esketamine-a non-psychedelic enantiomer of ketamine-became approved by the FDA as a psychiatric treatment for major depressive disorder [7]. Currently, MDMA is estimated to be the next psychedelic to be FDA-approved potentially. Psilocybin, ketamine, and MDMA all have an extensive history of use in recreational settings. However, in controlled clinical settings, each has been adapted for therapeutic purposes as an adjunct in treatment [8].

When paired with therapy, MDMA is the only psychedelic drug proven efficacious for treatment-resistant post-traumatic stress disorder (PTSD). Methyl​enedioxy​methamphetamine-assisted therapy (MDMA-AT) to treat other conditions such as eating disorders and alcohol use disorder continues to be researched. To date, MDMA-AT is found to be potentially beneficial for various problems related to psychological trauma, such as anxiety from a life-threatening illness and social anxiety in autistic adults.

History of MDMA

In 1912, pharmaceutical company Merck filed two patent applications that described the chemical synthesis of MDMA and its unique psychoactive properties. Further drug development research halted until 1959 when Wolfgang Fruhstorfer synthesized MDMA for pharmacological testing while researching stimulants [9]. In the 1960s, chemist Alexander Shulgin documented his experiments with synthesizing MDMA and numerous other related compounds in *Phenethylamines I have known and loved (PIHKAL): A Chemical Love Story *[9]. Leo Zeff would later use MDMA as an adjunct to psychotherapy, introducing it to approximately 150 other psychotherapists, ultimately responsible for an estimated 500,000 doses of MDMA administered in therapeutic settings [10]. By the 1980s, many psychotherapists adapted MDMA as part of their practice due to its ability to accelerate the therapeutic process. Although there were no clinical trials nor government approval, MDMA-AT was legally used for couples counseling, relationship problems, depression, anxiety, substance use disorders, premenstrual syndrome, and autism, among several other psychiatric disorders [10].

As the war on drugs raged on with racially disproportionate mass incarceration amid crack cocaine-dominated headlines, MDMA raised the attention of the Drug Enforcement Agency (DEA). During a debate on the Phil Donahue show on banning MDMA, researchers shared pre-published reports stating that the drug caused brain damage in rats and so it could do the same in humans. However, the information was misleading since it did not note the significantly large and frequent intravenous doses given to the rats [11]. A study [12] published in Science showing MDMA was neurotoxic in primates was later redacted due to the lab being found to have been injecting methamphetamine instead of MDMA [13]. Other news sources furthered the claim that MDMA caused brain damage, but they confused it with the ‘synthetic heroin’ designer drug 1-methyl-4-phenyl-1,2,3,6-tetrahydropyridine (MPTP), which caused acute onset Parkinsonism. Methyl​enedioxy​methamphetamine entered a burgeoning recreational drug counterculture before being banned as a Schedule I substance in 1985 as another salvo in the War on Drugs [10].

The DEA’s drug denominations range from Schedule I to V, with Schedule I drugs having the highest risk of abuse and Schedule V drugs having the lowest potential for abuse [14]. The DEA designated MDMA as Schedule I, declaring that it had “no currently acceptable medical use” [14] despite the Schedule III recommendation from the DEA judge overseeing the proceedings [15]. Due to the extensive hurdles involved with DEA approval and public funding, clinical research was stunted. Medical researchers still believe in MDMA's therapeutic potential, particularly among people with PTSD, depression, and other psychiatric issues. In 1986, the nonprofit Multidisciplinary Association for Psychedelic Studies (MAPS) was founded to secure private funding for clinical trials despite these barriers [10]. Since then, MAPS has raised over $130 million for scientific research of psychedelics and cannabis for therapeutic purposes. In 2004, the DEA and the FDA approved MAPS to research MDMA's efficacy as an adjunct to psychotherapy. The first clinical trial of MDMA-AT was conducted in Spain but ended early due to political pressure [16]. The first completed study was conducted in the United States and was published in 2011 [17].

## Review

Clinical trials

The FDA granted breakthrough therapy status to MDMA-AT in 2017 based on evidence from the phase-II clinical trials [18]. Multidisciplinary Association for Psychedelic Studies reported a series of six phase-II clinical trials looking at MDMA-AT for PTSD. Participants met the Diagnostic and Statistical Manual of Mental Disorders, 4th Edition, Text Revision (DSM-4-TR) criteria for a diagnosis of PTSD and were treatment-resistant to at least three months of antidepressant treatment and six months of psychotherapy. Participants were provided several preparatory pre-MDMA sessions, two or three eight-hour MDMA-assisted psychotherapy sessions each one month apart, and several follow-up non-drug psychotherapy sessions described as “integration” sessions. After two MDMA-AT sessions, 54% of participants in the MDMA group did not meet the criteria for PTSD compared to 23% of the placebo group [19]. After three MDMA-AT sessions, 67% of participants no longer met the criteria for PTSD [20]. These effects were durable at the one-year follow-up, with 67% not meeting PTSD criteria [21]. At nearly four years, 74% no longer met PTSD criteria. Participants who reported suicidal ideation decreased from approximately 60% to 24% [21].

In 2021, MAPS and the FDA agreed on the design for phase-III trials. The phase -III trials were the first multi-site randomized, double-blinded, placebo-controlled trial, testing 90 participants with severe PTSD [20]. Participants received three sessions with 80 mg to 120 mg of MDMA and either an additional half-dose after 1.5-2 hours or, a placebo combined with three preparatory and three integrative therapy sessions after each medication session. Drug safety included assessing the participant's heart rate, blood pressure, and body temperature while being cognizant of any signs of adverse events and thoughts such as suicide. Common side effects included decreased appetite, muscle tightness, and hyperhidrosis, but they were transient and mild to moderate in severity [20].

Phase-III data indicates that MDMA-AT is highly effective, safe, and well-tolerated in individuals who have a treatment-resistant PTSD diagnosis, and even in those with comorbidities. Post-traumatic stress disorder treatments are desperately needed, and MDMA-AT represents a potential breakthrough treatment that merits expedited clinical development [20].

A second phase-III clinical trial at MDMA-AT-approved clinics is currently enrolling participants and is expected to be published in 2022. If the FDA approves MDMA-AT, policies will be required for determining physician qualifications to prescribe and administer MDMA, MDMA-AT site qualifications, and how it will be produced and stored.

Neuropsychological effects of MDMA

Methyl​enedioxy​methamphetamine is a psychoactive drug that increases the endogenous release of the neurotransmitters serotonin, norepinephrine, and dopamine. It also leads to the release of neurohormones such as oxytocin, which is thought to be a primary mediator of its effects. Subjective effects include stimulating social connectedness, empathy, euphoria, and feelings of communion. Oxytocin has emerged as an essential component in elucidating the reopening of critical periods for social reward learning and mediating interpersonal bonding [22]. The use of MDMA alongside psychotherapy allows participants to revisit past distressful memories in a state of emotional security and empathic self-reflection.

Individuals with PTSD have an amplified and uncontrolled response from the amygdala to trauma-specific cues. Increasing serotonin helps regulate mood, while oxytocin increases trust and emotional awareness by reducing the amygdala’s response [23]. The effects are decreased hypervigilance and anxiety and improved states of consciousness [24]. Moreover, MDMA activates serotonin (5-HT2A) receptors, which increases sedation and relaxation that may be conducive to addressing trauma-induced hypervigilance and promoting memory consolidation [25].

Oxytocin, commonly referred to as the ‘love hormone,’ has sparked the interest of scientists for its potential to regulate anxiety and promote social bonding. However, translational pharmaceutical research has proven difficult, as oxytocin does not readily cross the blood-brain barrier. Methyl​enedioxy​methamphetamine is an indirect inducer of oxytocin release and thus may explain MDMA's effects on PTSD. Because of this unique property, Nardou et al. [22] decided to use MDMA to investigate oxytocin's role in critical periods. Critical periods are developmental periods during which the nervous system is expressly sensitive to specific environmental stimuli required for proper organization and learning [22]. Closure of critical periods limits the ability of the brain to adapt even when optimal conditions are restored. This could explain the high treatment non-response rates seen in PTSD. A single dose of MDMA was enough for adult mice to reopen the critical period for social reward learning via oxytocin-mediated plasticity in the nucleus accumbens. In contrast, isolated adult mice administered MDMA did not exhibit the reopening of their critical period. These data suggest that it is the combination of the MDMA-mediated effects of oxytocin along with a therapeutic environment that is the key to the therapeutic efficacy of MDMA-AT [22].

MDMA-AT process

The treatment approach of MDMA-AT is ultimately an interaction between the drug's effects, the therapeutic setting, and the mindset of the participant and therapists. Methyl​enedioxy​methamphetamine catalyzes therapeutic processing, promoting participants’ emotional engagement with decreased anxiety or other painful emotions at the time of revisiting traumatic experiences. Frequently, participants can experience and express fear, anger, grief, empathy, love, and gratitude as part of the therapeutic process in a manner with less judgment and greater acceptance. Also, MDMA can facilitate a heightened state of empathic therapeutic rapport that promotes the therapeutic process and develops a corrective experience of secure attachment [8].

The primary focus of MDMA-AT is to diminish symptoms related to unresolved trauma and improve the overall wellbeing and quality of life of the participant. Processing traumatic experiences is an essential part of MDMA-AT, though sessions may explore other psychological, interpersonal, and spiritual aspects of life. Empathy and self-exploration facilitate the healing of participants since it allows each participant's experience to unfold spontaneously. However, if a patient encounters emotional or somatic blocks that stymie this process, the therapist can provide more active guidance to help them work through past events and arrive at a new emotional resolution. Therapists help explore and validate new perspectives about other life experiences and authentically join participants in embracing joyful moments [26].

The MDMA-AT consists of a preparatory stage with screening and introductory sessions, followed by one to three experimental sessions interspersed with integrative sessions and follow-up evaluations. The screening and preparatory stage is when the therapist gathers the participant’s history and builds therapeutic rapport [27]. During the experimental stage, an 80 mg to 120 mg capsule of MDMA is orally administered. The participant is monitored through medical devices, listens to a predetermined setlist of emotionally provocative music, engages in mindful use of touch as appropriate, and engages in conversation with the therapist. Peak effects typically occur 70 to 90 minutes after drug administration and persist for one to three hours [28]. After two hours, a supplemental half dose of 40 mg to 60 mg is optionally provided. Therapists work with the participant for six to eight hours or until the drug's psychedelic effects have worn off. As the MDMA subsides, therapists may talk with the participant more extensively about what they experienced during the session [27].

During the MDMA experience, participants acquire heightened clarity about the traumatic event and can view it as something of the past. Participants have disclosed that through MDMA-AT, processing painful emotions successfully changed their relationship with their emotions and trauma narrative. Methyl​enedioxy​methamphetamine grants access to meaningful spiritual experiences and other transpersonal experiences. Although unexplainable, many participants feel a sense of healing on a non-verbal level, which is deemed essential to the therapeutic process [29].

Stigma surrounding MDMA-AT

The terms 'ecstasy' and 'MDMA' are commonly confused. Substances classified as ecstasy may contain MDMA, but frequently contain other unknown and/or dangerous components. In controlled doses, pure MDMA has been proven safe for human consumption. Furthermore, critics highlight studies that illustrate the dangers of MDMA as a recreational drug, which is misleading since there are sufficient differences between ecstasy and MDMA that would confound a generalized comparison between the two [30].

While it is well established in rat studies that injected doses of MDMA are neurotoxic, these doses are well above those provided for therapy. Likewise, human trial participants have failed to show evidence of neurotoxic effects of any minuscule amount. Additionally, while some studies have shown an inconsistent pattern of mild memory deficits, these have been blended by substantial polysubstance use among MDMA users [11]. In a study of 87 deaths where MDMA was present, only six involved the drug alone, and the two most common causes of death were heatstroke and anti-diuretic hormone (ADH)-mediated hyponatremia, both likely exacerbated by recreational settings with increased physical exertion [31]. Of the approximately 200 participants in recent clinical trials of MDMA-AT for PTSD, none have suffered serious adverse events related to hyponatremia or hypothermia, which are the most common causes of morbidity and mortality in recreational settings [20].

Yet, MDMA is to some degree unpredictable, producing diverse responses in people. Methyl​enedioxy​methamphetamine causes neurotransmitter activation across the main neural pathways, including serotonin and dopamine, and norepinephrine, resulting in substantial fluctuations in mood and emotions depending on the memories that emerge for the participant. When the effects of MDMA taper, there is a neurochemical depletion stage due to serotonin exhaustion. Neurochemical depletion can invoke temporary anhedonia, lethargy, anger, depression, irritability, anxiety, increase in daily stress, altered pain thresholds, changes in sleep, and nightmares, especially in female participants [32]. However, these pitfalls are duly addressed in MDMA-AT by having the participant stay overnight at the site of administration with a trained same-sex overnight attendant, and the first integration session is scheduled for the morning after with the second integration session often within a week after the MDMA session to allow for closer monitoring and follow-up care.

Methyl​enedioxy​methamphetamine has a risk of dependence and harm compared to other recreational substances [33,34] and is often described as a "self-limiting" drug due to its usage patterns often being relatively infrequent [35]. Because MDMA is a self-limiting drug, dependency rates may be as low as 1% of users [24].

## Conclusions

Clinical trials have found MDMA-AT to be safe and effective for PTSD treatment, and preliminary evidence suggests its use in the 1980s for other psychiatric conditions may indeed be founded. The final phase-III study is nearing completion in 2022, and FDA approval is estimated for 2023. Hawaii has a great need for access to effective treatment options for PTSD and other psychiatric conditions. The aim of Hawaii’s Senate Bill 738 to create access for psilocybin treatment needs to be expanded to also encompass MDMA-AT as a treatment option. Considering the weight of the current state of evidence for the safety and efficacy of MDMA-AT, an approach of evidence-based policy would necessitate that this treatment option is made available to those who need it in the state of Hawaii.
